# Analysis of the psychological impact of a vascular risk factor intervention: results from a cluster randomized controlled trial in Australian general practice

**DOI:** 10.1186/1471-2296-14-190

**Published:** 2013-12-13

**Authors:** Suzanne Helen McKenzie, Upali W Jayasinghe, Mahnaz Fanaian, Megan Passey, Mark Fort Harris

**Affiliations:** 1 Centre for Primary Health Care and Equity, School of Public Health and Community Medicine, University of New South Wales, Kensington, NSW, Australia; 2 School of Medicine and Dentistry, James Cook University, Townsville, Queensland, Australia; 3 University Centre for Rural Health – North Coast, School of Public Health, University of Sydney, Darlington, NSW, Australia; 4 Illawarra Health and Medical Research Institute, University of Wollongong, Wollongong, NSW, Australia

## Abstract

**Background:**

Screening for vascular disease, risk assessment and management are encouraged in general practice however there is limited evidence about the emotional impact on patients. The Health Improvement and Prevention Study evaluated the impact of a general practice-based vascular risk factor intervention on behavioural and physiological risk factors in 30 Australian practices. The primary aim of this analysis is to investigate the psychological impact of participating in the intervention arm of the trial. The secondary aim is to identify the mediating effects of changes in behavioural risk factors or BMI.

**Methods:**

This study is an analysis of a secondary outcome from a cluster randomized controlled trial. Patients, aged 40–65 years, were randomly selected from practice records. Those with pre-existing cardiovascular disease were excluded. Socio-demographic details, behavioural risk factors and psychological distress were measured at baseline and 12 months. The Kessler Psychological Distress Score (K10) was the outcome measure for multi-level, multivariable analysis and a product-of-coefficient test to assess the mediating effects of behaviour change.

**Results:**

Baseline data were available 384 participants in the intervention group and 315 in the control group. Twelve month data were available for 355 in the intervention group and 300 in the control group. The K10 score of patients in the intervention group (14.78, SD 5.74) was lower at 12 months compared to the control group (15.97, SD 6.30). K10 at 12 months was significantly associated with the score at baseline and being unable to work but not with age, gender, change in behavioural risk factors or change in BMI.

**Conclusions:**

The reduction of K10 in the intervention group demonstrates that a general practice based intervention to identify and manage vascular risk factors did not adversely impact on the psychological distress of the participants. The impact of the intervention on distress was not mediated by a change in the behavioural risk factors or BMI, suggesting that there must be other mediators that might explain the positive impact of the intervention on emotional wellbeing.

**Trial registration:**

Australian New Zealand Clinical Trials Registry ACTRN12607000423415.

## Background

Behavioural and physiological risk factors for vascular disease are common in patients presenting to Australian general practitioners [1] and intensive lifestyle interventions have been shown to prevent vascular disease and diabetes in high risk patients [2-4]. Screening for vascular disease and risk assessment is encouraged in general practice [5] however there is limited evidence about the emotional impact. A recent systematic review and meta-analysis concluded that there are few long term adverse emotional impacts of screening and risk assessment however it included only one study about cardiovascular risk [6].

Lifestyle modification programs encourage participants to make significant changes through education, support and self-management strategies [7,8]. Unless specific psychological interventions were included in the programs the emotional impacts of these programs have not been measured. As there is some evidence that positive behavioural changes such as increasing physical activity result in improved mental health in those diagnosed with depression or anxiety disorders [9], the assumption has been that there would be no adverse emotional consequences for participants in trials of intensive lifestyle interventions. Psychological stress may be helpful in some circumstances but too much distress can reduce quality of life and the ability to make informed choices regarding treatment options [10]. Higher levels of distress for example, reduce the probability of meeting recommended levels of physical activity [9].

The Health Improvement and Prevention Study was a cluster randomized controlled trial of vascular risk factor management in Australian general practice [8,11]. It evaluated the impact of a general practice-based intervention for patients at risk of vascular disease on behavioural and physiological risk factors in 30 practices. The intervention consisted of risk factor identification and referral to a lifestyle modification program. The current study was embedded in the HIPS trial and used validated measures to assess the psychological effects of the intervention.

Specifically this study aimed to 1. Investigate the psychological impact of participating in the intervention arm of the trial of vascular risk factor management and; 2. Identify the mediating effects of changes in behavioural risk factors (diet, physical activity, cigarette smoking and alcohol intake) or BMI. The study hypotheses were that patients who participated in the intervention arm of the trial would report lower psychological distress at 12 months, compared to patients in the control group; and that the reduction in psychological distress would be mediated by an improvement in one or more of their behavioural risk factors.

## Methods

The Health Improvement and Prevention Study (HIPS) was a stratified cluster randomized controlled trial conducted in general practices in New South Wales, Australia (Australian New Zealand Clinical Trials Registry, ACTRN12607000423415). The methodology and outcomes of the trial have been described previously [8,11].

### Recruitment

The trial involved 30 practices who used electronic medical records and had expressed interest in the trial. Sixteen practices were randomly allocated to the study intervention group and 14 to the study control group. Patients were eligible to participate in the study if they had attended the practice in the preceding 12 months and were either aged 40–55 years with a diagnosis of hypertension and/or hyperlipidaemia or were aged 56–64 years with or without recorded risk factors. Exclusion criteria included diabetes, cardiovascular disease, current severe illness, inability to speak adequate English or understand the consent form. The majority of general practice patients aged over 64 years have already developed chronic disease so were not included. Each practice randomly selected up to 160 patients from practice records and invited them by mail to have fasting blood sugar and lipids levels; and attend their practice for a health check at baseline and 12 months.

### Intervention

The patients in the intervention practices attended for a structured health check visit. During the visit practice staff who had received prior training, assessed their vascular risk factors (blood pressure, lipids, fasting blood glucose, body mass index, waist circumference, smoking, nutrition, alcohol intake, and physical activity) and provided brief lifestyle advice and motivational interviewing. The brief intervention was modeled on the 5As framework (ask, assess, advise, assist and arrange) [12]. Patients were referred to the lifestyle modification program if they were found to be at high risk [8]. Sixty-three percent of the 301 eligible patients were referred to the program [13]. The lifestyle program was provided in the local area of each participating practice and coordinated by a program manager. It consisted of one individual visit with a dietitian or exercise physiologist for assessment and individual goal setting, followed by four, 1.5 hour, group sessions over 3 months; and a further two follow-up sessions at 6 and 9 months. The group sessions were adapted from the “*Counterweight Program- CHANGE*” [14] and included education, physical activity and self management strategies (goal setting, self monitoring, developing practical skills and problem solving) aimed at promoting positive dietary and physical activity changes, reduction in alcohol intake, smoking cessation and weight loss.

Patients attending the control practices were also invited to attend the practice for a routine health check. At this they received usual general practice care for their risk factors by general practitioners who had not received the prior training.

### Data collection

Patients, who were blinded to the practice allocation, were mailed a questionnaire at baseline and 12 months and asked to complete it in private before attending their practice for the health check. The questionnaire, based on previous research, collected self-reported behaviours [15] (baseline and 12 months), demographic characteristics [15] (baseline) and the Kessler Psychological Distress Scale [16] (baseline and 12 months).

Self-reported behaviours included:

• Current smoking status [5]

• Serves of fruit and vegetables per day (diet risk, <7 serves per day) [17]

• Alcohol consumption (number of standard drinks on a typical day) [5]

• Physical activity level (included duration of vigorous and moderate physical activity) (score range 0–8, Inactivity, <4) [18]

Demographic information included gender, age and markers of socioeconomic status (home ownership and employment status) [19]. Self-reported employment categories were full time employment, full time education, unemployed, unable to work, looking after family, retired or other. Self-reported home ownership categories were living in own home, living in rented accommodation, other living arrangements.

The Kessler Psychological Distress Scale (K10) [16] is a ten item questionnaire measuring negative emotional states in the preceding four weeks. Responses are rated on a five point scale and summed to produce a score from 10 to 50. High scores (30–50) are strongly associated with a diagnosis of a psychiatric disorder. The instrument is used in clinical practice and is sensitive to changes resulting from interventions [20,21].

Body Mass Index (BMI) was calculated using height and weight from audits of medical records at baseline and 12 months. A participant was considered “at risk” if their BMI ≥ 25 kg/m^2^[5].

### Randomization

A statistician who was not involved in the data collection used computer generated random numbers to randomly allocate practices to intervention and control groups, stratified by location. Data collection officers were blinded to the allocation of practices.

### Sample size calculation

A priori sample size calculation for the secondary analysis on the K10 score confirmed that 350 patients in each group would have 80% power and 5% significance level to detect an effect size of 0.28 between intervention and control groups at 12 months assuming 15% lost to follow-up after adjustment for clustering (ICC = 0.025) [22].

The effect size was based on expert opinion. Larger effect sizes have been demonstrated in groups with a diagnosis of psychiatric disorder undergoing specific therapy but this population did not represent the participants in our study [21].

### Statistical analyses

We conducted bivariate analyses using ANCOVA or Chi-square (for categorical data) to test for differences between intervention and control groups at 12 months, using SPSS, version 15 (SPSS Inc) [23]. An intention to treat analysis was conducted including those lost to follow up (dropouts) if data were available and patients had not requested withdrawal of their data. Missing data was not included and no data was imputed. Multilevel analysis allows for incomplete outcome data as long as a missing at random process can be assumed [24]. The characteristics of the dropouts were compared with other participants at baseline. Multilevel multivariable analysis using MLwiN (statistical software for multilevel models) [25] was conducted for the sub group of participants who had complete K10 data at 12 months using list-wise deletion of missing values. Patients (level 1) were clustered within general practices (level 2). Initially, we fitted a baseline variance component model (no independent variables) for K10 at 12 months followed by the main model. The main multilevel model added covariates and included patients’ gender, age, home ownership status and employment.

Change in diet, BMI, physical activity and alcohol were examined as potential mediators of the lifestyle intervention effect on a change in psychological distress after adjustment for age, home ownership status and employment. Change variables were computed using the formula: 12 month score – baseline score. Smoking status was categorical and a change score was not computed. Therefore it was not included in the subsequent analysis.

To assess mediating effects, a product-of-coefficient test, appropriate for cluster randomized controlled trials, was used [26]. This test consists of (1) estimating the effect of the intervention on changes in the behavioural mediator (α coefficient) by regressing changes in the mediator onto the intervention; (2) estimating the independent effect of changes in the potential mediator on changes in psychological distress (β coefficient) by regressing changes in psychological distress onto the intervention and changes in the mediator; (3) computing the product of the two coefficients αβ, representing the mediated effect; (4) dividing αβ by its standard error. The estimates were obtained using multilevel linear regression models (ICC for the multilevel mediator model was 0.016), accounting for age, home ownership, employment and within-practice cluster effects. The ratio of the total mediated effect to total intervention effect was also estimated. The standard error of the mediated effect was computed using the multivariate delta method [27]. All mediation analyses were conducted using MLwiN and Microsoft Excel.

### Ethics

Ethics approval for the study was obtained from the University of New South Wales (UNSW) Human Research and Ethics Committee. All participants gave fully informed written consent.

## Results

**Figure 1:**
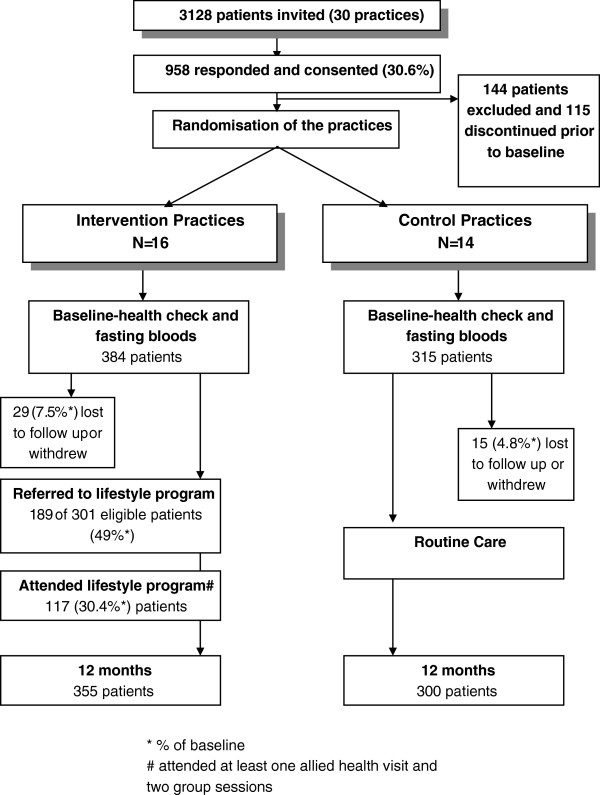
Participant recruitment, flow and follow-up.

A total of 3128 patients from the 30 participating practices were approached to participate in the study and 958 (30.6%) consented. A higher number of patients from the intervention practices consented. Following exclusions and withdrawals baseline data were available for 699 participants (384 in the intervention group and 315 in the control group) and following loss to follow up, 12 month data for up to 655 participants (355 in the intervention group and 300 in the control group) [8]. Those who were lost from the study were more likely to be overweight (P = 0.04) but were otherwise similar to those completing the study. Figure 1 illustrates the recruitment and flow of participants through the HIPS trial.

### Baseline characteristics

Table 1 shows the characteristics of the respondents at baseline. Of the 699 participants, 401 (57.4%) were female, 525 (75.1%) were in the older age group (56–64 years), 326 (46.6%) were tertiary educated and 479 (68.5%) employed. 114 (16.3%) had one behavioural risk factor, 247 (35.3%) had two and 316 (45.2%) had three or more behavioural risk factors. The mean K10 score (Cronbach alpha = 0.896) for the sample was 16 (SD 6.3). There were no significant differences at baseline in the characteristics of intervention and control patients.

**Table 1:** Baseline characteristics of intervention and control patients at baseline (n = 699)

	**Intervention (n = 384)**	**Control (n = 315)**
**Demographic characteristics**	**Number (%)**	**Number (%)**
Female	232 (60.4%)	169 (53.7%)
Age		
40–55 years	96 (25.0%)	78 (24.8%)
56–64 years	288 (75.0%)	237 (75.2%)
Tertiary educated	173 (45.1%)	153 (48.6%)
Employed	254 (66.1%)	225 (71.4%)
Own accommodation	308 (80.2%)	249 (79%)
**Risk factors**		
Body mass index ≥25	229 (59.6%)	166 (52.7%)
Portions of fruit and vegetables consumed per day <7	308 (80.2%)	263 (83.5%)
Physical activity score <4	209 (54.4%)	193 (61.3%)
Tobacco smoker	45 (11.7%)	43 (13.7%)
Alcohol intake >2 standard drinks per day	120 (31.3%)	99 (31.4%)
**Psychological distress**	**Average score (SD)**	**Average score (SD)**
K10 Score	15.61 (5.73)	15.77 (5.71)

### Bivariate analysis

The K10 score of the patients in the intervention group reduced between baseline and 12 months indicating lower distress, while the K10 score in the control group remained unchanged. There was a significant difference in K10 at 12 months between the intervention and control groups (F = 11.43, p = 0.001) (Table 2).

**Table 2:** Patient outcomes for intervention and control groups at baseline and 12 months

	**Intervention**		**Control**		**ANCOVA for intervention and control groups at 12 months**
**Patient outcome**	**Baseline**	**12 Months**	**Baseline**	**12 Months**	
	**N = 384**	**N = 355**	**N = 315**	**N = 300**	
	**Mean (SD)**	**Mean (SD)**	**Mean (SD)**	**Mean (SD)**	
**K10**	15.67 (5.73)	14.78 (5.74)	15.77 (5.69)	15.97 (6.30)	F = 11.43, p = 0.001
**BMI**	28.97 (5.58)	28.06 (5.07)	29.68 (6.90)	28.39 (5.96)	NS
**Serves of fruit and vegetables**	4.73 (2.12)	4.85 (2.82)	4.59 (2.08)	4.52 (2.59)	NS
**Physical activity score**	3.71 (2.38)	4.60 (2.49)	3.38 (2.40)	4.09 (2.48)	NS
**Alcohol intake**	1.63 (0.97)	1.5 (0.80)	1.62 (0.94)	1.60 (0.95)	NS
**Tobacco smoker**	45 (11.7%) (8.5%–14.9%)	30 (9.6%) (6.3%–12.8%)	43 (13.7%) (9.9%–17.4%)	31 (11.6%) (7.8%–15.5%)	NS*

*Chi- square test.

There was no significant difference between intervention and control groups at 12 months for BMI, daily intake of fruit and vegetables, physical activity, smoking or alcohol intake (Table 2).

### Multilevel analysis

We examined the association between the intervention and K10 at 12 months using multilevel multivariable linear regression adjusted for patient characteristics and cluster effects. The K10 score of patients allocated to the intervention group was lower compared to the control group (Table 3). K10 score at baseline and being unable to work were significantly associated with K10 at 12 months.

**Table 3:** Multilevel linear regression model for K10 at 12 months*

**Parameters**	**Baseline model**		**Estimate for main model**		**P value**
	**B (SE)**	**95% CI**	**B (SE)**	**95% CI**	
Intercept	15.276		5.799		
Intervention			-1.137(0.392)	-1.905, -0.369	P <0.01
Baseline K10			0.611(0.035)	0.542, 0.680	P < 0.001
Unable to work			3.867(0.890)	2.123, 5.611	P < 0.001
Variance between practices	1.850(0.993)	0.00, 3.796	0.044(0.259)	0.00, 0.552	
Variance between patients	32.876(2.063)	28.833, 36.919	19.068(1.191)	16.734, 21.402	

*After list-wise deletion of missing values 536 patients and 30 practices were used in the analysis.

Note: B = regression coefficient; SE = standard error.

K10 at 12 months was not associated with the other markers of socioeconomic status, age, gender, change in any of the behavioural risk factors or change in BMI.

### Mediation analysis

The change in K10 in the intervention group was not associated with change in any of the behavioural risk factors or BMI in the multiple-mediator model (Table 4).

**Table 4:** Single and multiple multilevel mediator models for the association between intervention and change in distress (K10) controlling for age, home ownership and employment

	**Single –mediator model**			**Multiple-mediator model**		
	**αβ (SE)**	**95% CI of αβ**	**Z (P)**	**αβ (SE)**	**95% CI of αβ**	**Z (P)**
Change in:						
Diet score	-0.137 (0.075)	-0.284,0.010	-1.833 (0.066 )	-0.172 (0.089)	-0.346,0.003	-1.930 (0.053)
BMI	0.014 (0.024)	-0.034,0.061	0.568 (0.570)	-0.008 (0.019)	-0.045,0.029	-0.405 (0.686)
Physical activity score	-0.007 (0.018)	-0.042,0.028	-0.388 (0.698)	-0.013 (0.025)	-0.063,0.037	-0.504 (0.614)
Alcohol score	-0.027 (0.035)	-0.096,0.043	-0.749 (0.454)	-0.019 (0.031)	-0.079,0.041	-0.623 (0.534)

Notes: Multilevel regression models were adjusted for age, home ownership, employment and within-practice clustering effects.

α estimate of intervention effect on change score of behavioural factors.

β estimate of the independent effect of the change mediator score on change K10 score (controlling for intervention).

αβ (mediated effect) product-of-coefficient estimate.

SE standard error.

95% CI of αβ 95% confidence interval of the mediated effect.

z standard deviate associated with mediated effect (used for significance testing).

## Discussion

This analysis of a secondary outcome from a cluster randomized controlled trial of a general practice based health check to identify vascular risk factors, with referral of at risk patients to a lifestyle modification program demonstrated that the intervention reduced the psychological distress of the participants. The only primary outcome associated with the intervention, using multi-level, multi-variable analysis on an intention to treat basis and adjusted for patient and practice characteristics, time and cluster effects, was an increase in self-reported physical activity [8]. There was also a small weight reduction (1.06 kg) only among those attending the group program [8]. However the reduction in psychological distress was not mediated by a change in the behavioural risk factors or BMI in the multi-mediator model. This finding is in contrast to other studies in which an increase in physical activity and weight loss has been shown to reduce symptoms of anxiety and depression [9].

Few previous intervention studies for the primary prevention of vascular disease have identified the emotional impact of their interventions. The reduction in psychological distress in this study was small however similar to other studies where the intervention was not specifically designed to reduce distress [28,29]. The distress level of the participants was already low and importantly the intervention did not increase their distress. Achieving goals and possibly improving self- efficacy in relation to behavioural choices may have contributed to the reduction in distress. Self-efficacy is an important factor in successful behaviour change [30] however the relationship between this and distress has not been explored.

Other studies have demonstrated a short term (4–6 weeks) increase in anxiety following screening but no longer term increase [6,31]. We were unable to measure any short term change in distress due to the limitations of the trial protocol and by measuring distress at 12 months we missed any possible short term increase. However the intervention included risk factor screening and a health check delivered by GPs and practice nurses who had been trained in motivational interviewing as well as a lifestyle modification program for those who were identified with one or more vascular risk factors. While the health check or lifestyle modification program did not address stress or mental health specifically, the social support from the practice team and/or group members may have helped to reduce any individual distress in the participants. It is also possible that the self management skills covered during the group program and/or the motivational interviewing from the initial health check may have assisted individuals to reduce any distress resulting from risk factor awareness. Unfortunately we did not measure each sub-component of the intervention and were therefore unable to identify which contributed to the impact on distress. We were also not able to sub- group analysis on the 30.4% of the participants in the intervention group who attended the lifestyle program as we did not have adequate power to determine whether the group program had more or less impact on the distress levels of attendees compared to those who did not attend. These are limitations of this study and require further exploration through other studies.

We included socio-demographic variables in the analysis as these have been strongly associated with psychological distress [32,33] but in contrast to our baseline study we found an association between being unable to work and distress rather than between being unemployed and distress. The reasons for this is unclear but it may be that not being able to fulfill a desire, in this case to work, is the underlying reason for the association rather than the socio-economic factors [34].

This study was designed to be conducted in conjunction with the primary HIPS study. The limitations of the HIPS study have already been reported [8]. Self- reported data used in the study could have had significant response bias as the participants were aware that they were in a study. However this should have affected both intervention and control groups and not changed the findings of the study. The actual ICC computed from the main multilevel model was 0.002 which is much lower than that used in the power calculations. Using this ICC, 260 patients from each group was required to detect changes in psychological distress. Therefore the study was powered to detect changes in psychological distress. The characteristics of the participants who were lost to follow-up were similar to those remaining in the study except they were more likely to be overweight. However, due to missing values only 536 participants were included in the multi-level linear regression model. It is possible that those with missing values had a higher distress level compared to those who completed the questionnaire, therefore biasing the result towards a lower K10 score at 12 months.

Our findings may not be generalisable to all general practices as a limited sample was chosen and only those interested responded to the invitation. Individual practices were randomized to intervention and control groups and it is unlikely that practitioners in the intervention group communicated with the control practices. The intervention was monitored by study facilitators and every attempt was made to ensure its fidelity. The patients were randomly chosen from practice records and their demographic characteristics, baseline behavioural risk factors and psychological distress were comparable to the general population [35,36].

## Conclusion

Australian general practice has an important role in screening for vascular risk factors and providing appropriate interventions. This study confirms that this process does not adversely impact on the psychological distress of our patients. Changes in behavioural risk factors or BMI did not mediate the intervention’s impact on distress. There are a range of other potential mediating factors, including social support, that require further exploration.

## Abbreviations

ANCOVA: Analysis of covariance; BMI: Body mass index; HIPS: Health improvement and prevention study; ICC: Intraclass correlation coefficient; K10: Kessler psychological distress scale; SD: Standard deviation; UNSW: University of New South Wales.

## Competing interests

The authors declare that they have no competing interests.

## Authors’ contributions

SM designed the study, conducted data analyses, interpreted the results and drafted the manuscript. UJ performed analysis and drafted the manuscript. MF coordinated the study and acquired the data. MP interpreted the results and drafted the manuscript. MH designed the study and interpreted the results. All authors have contributed to drafting and revising the manuscript; and approved the final manuscript.

## Pre-publication history

The pre-publication history for this paper can be accessed here:

http://www.biomedcentral.com/1471-2296/14/190/prepub

## References

[B1] Britt H Miller GC Charles J Henderson J Bayram C Pan Y Valenti L Harrison C Fahridin S O’Halloran J General practice activity in Australia, 2008–09. General practice series no. 25. GEP 25 2009 Canberra: AIHW [ http://www.aihw.gov.au/publication-detail/?id=6442468308] Accessed July 2012

[B2] Eriksson MK Franks PW Eliasson M A 3-year randomized trial of lifestyle intervention for cardiovascular risk reduction in the primary care setting: the Swedish Bjorknas study PloS One 2009 14 e5195 10.1371/journal.pone.0005195 19365563 PMC2664964

[B3] Ebrahim S Beswick A Burke M Davey Smith G Multiple risk factor interventions for primary prevention of coronary heart disease Cochrane Database Syst Rev 2006 1 CD001561 doi:10.1002/14651858.CD001561.pub3 17054138 10.1002/14651858.CD001561.pub2 PMC4160097

[B4] Tuomilehto J Lindstrom J Eriksson JG Valle TT Hamalainen H Ilanne-Parikka P Keinanen-Kiukaanniemi S Laakso M Louheranta A Rastas M Salminen V Uusitupa M for the Finnish Diabetes Prevention Study, Group Prevention of Type 2 diabetes melllitus by changes in lifestyle among subjects with impaired glucose tolerance N Engl J Med 2001 14 1343 1350 10.1056/NEJM200105033441801 11333990

[B5] Royal Australian College of General Practitioners Guidelines for preventive activities in general practice 2012 8 East Melbourne [ http://www.racgp.org.au/download/Documents/Guidelines/Redbook8/redbook8.pdf] Accessed November 2012

[B6] Collins RE Lopez LM Marteau TM Emotional impact of screening: a systematic review and meta-analysis BMC Public Health 2011 14 603 http://www.biomedcentral.com/1471-2458/11/603 10.1186/1471-2458-11-603 21798046 PMC3223929

[B7] Counterweight Project Team and Trueman P Long-term cost-effectiveness of weight management in primary care Int J Clin Pract 2010 14 775 783 20353431 10.1111/j.1742-1241.2010.02349.x

[B8] Harris MF Fanaian M Jayasinghe UW Passey ME McKenzie SH Powell Davies G Lyle DM Laws RA Schutze H Wan Q A cluster randomised controlled trial of vascular risk factor management in general practice Med J Austr 2012 14 387 393 doi:10.5694/mja12.10313 10.5694/mja12.10313 23025735

[B9] Azevedo Da Silva M Singh-Manoux A Brunner EJ Kaffashian S Shipley MJ Kivimaki M Nabi H Bidirectional association between physical activity and symptoms of anxiety and depression: the Whitehall II study Eur J Epidemiol 2012 14 537 546 doi10.1007/s10654-012-9692-8 10.1007/s10654-012-9692-8 22623145 PMC4180054

[B10] Eich E Kihlstrom JF Bower GH Forgas JP Niedenthal PM Cognition and Emotion: Counterpoints: Cognition, Memory and Language 2000 Oxford: Oxford University Press

[B11] Fanaian M Laws RA Passey M McKenzie S Wan Q Powell Davies G Lyle D Harris MF Health improvement and prevention study (HIPS) - evaluation of an intervention to prevent vascular disease in general practice BMC Fam Pract 2010 14 57 doi:10.1186/1471-2296-11-57 10.1186/1471-2296-11-57 20687956 PMC2923104

[B12] Hung DY Shelley DR Multilevel analysis of the chronic care model and the 5A services for treating tobacco use in urban primary care clinics Health Serv Res 2009 14 103 127 10.1111/j.1475-6773.2008.00896.x 18783454 PMC2669639

[B13] Passey ME Laws RA Jayasinghe UW Fanaian M McKenzie S Powell Davies G Lyle D Harris MF Predictors of primary care referrals to a vascular disease prevention lifestyle program among participants in a cluster randomised trial BMC Health Serv Res 2012 14 234 doi10.1186/1472-6963-12-234 10.1186/1472-6963-12-234 22856459 PMC3483009

[B14] Counterweight Project Team Evaluation of the Counterweight Programme for obesity management in primary care: A starting point for continuous improvement Br J Gen Pract 2008 14 548 554 10.3399/bjgp08X319710 18682018 PMC2486382

[B15] Amoroso C Harris MF Ampt A Laws RA McKenzie S Williams AM Jayasinghe UW Zwar NA Powell Davies G Health check for 45–49 year old patients in general practice: feasibility and impact on practices and patient behaviour Aust Fam Physician 2009 14 358 362 19458808

[B16] Andrews G Slade T Interpreting scores on the Kessler Psychological Distress Scale (K10) Aust NZ J Public Health 2001 14 494 497 10.1111/j.1467-842X.2001.tb00310.x 11824981

[B17] National Health and Medical Research Council Dietary Guidelines for Australian Adults 2003 Canberra: Commonwealth of Australia [ http://www.nhmrc.gov.au/_files_nhmrc/publications/attachments/n33.pdf] (consulted May 2010)

[B18] Marshall AL Smith BJ Bauman AE Kaur S Reliability and validity of a brief physical activity assessment for use by family doctors Br J Sports Med 2005 14 294 297 10.1136/bjsm.2004.013771 15849294 PMC1725203

[B19] Jayasinghe UW Proudfoot J Barton CA Amoroso C Holton C Powell Davies G Beilby J Harris MF Quality of life of Australian chronically-ill adults: patient and practice characteristics matter Health Qual Life Outcomes 2009 14 50 19493336 10.1186/1477-7525-7-50 PMC2700088

[B20] Kessler RC Mroczek DK Measuring the effects of medical interventions Med Care 1995 14 Suppl 4 AS109 AS119 7723439

[B21] Perini SJ Slade T Andrews G Generic effectiveness measures: sensitivity to symptom change in anxiety disorders J Affect Disord 2006 14 123 130 10.1016/j.jad.2005.10.011 16337690

[B22] Adams G Gulliford MC Ukoumunne OC Eldridge S Chinn S Campbell MJ Patterns of intra-cluster correlation from primary care research to inform study design and analysis J Clin Epidemiol 2004 14 785 794 10.1016/j.jclinepi.2003.12.013 15485730

[B23] Van Breukelen GJP ANCOVA versus change from baseline had more power in randomized studies and more bias in nonrandomized studies J Clin Epid 2006 14 920 925 10.1016/j.jclinepi.2006.02.007 16895814

[B24] Goldstein H Multilevel Statistical Models 1995 London: Edward Arnold

[B25] Rashbash J Browne W Prosser B Multilevel analysis with MLwiN Software: A user’s guide to MLwiN version 2.0 2005 Bristol: Centre for multilevel modelling, University of Bristol

[B26] Krull JL MacKinnon DP Multilevel modeling of individual and group level mediated effects Multivariate Behav Res 2001 14 249 277 10.1207/S15327906MBR3602_06 26822111

[B27] MacKinnon DP Rose JS, Chassin L, Presson CC, Sherman SJ Contrasts in multiple mediator models Multivariate applications in substance use research 2000 Mahwah, NJ: Lawrence Erlbaum Associates Inc 141 160

[B28] Ackerman IN Graves SE Bennell KL Osborne RH Evaluating quality of life in hip and knee replacement: Psychometric properties of the World Health Organization Quality of Life short version instrument Arthritis Rheum 2006 14 583 590 10.1002/art.22107 16874780

[B29] Sawyer MG Frost L Bowering K Lynch J Effectiveness of nurse home-visiting for disadvantaged families: results of a natural experiment BMJ Open 2013 14 e002720 doi:10.1136/bmjopen-2013-002720 23619089 10.1136/bmjopen-2013-002720 PMC3641507

[B30] Bandura A Self-efficacy: toward a unifying theory of behaviour change Psychol Rev 1977 14 191 215 847061 10.1037//0033-295x.84.2.191

[B31] Park P Simmons RK Prevost AT Griffin SJ Screening for type 2 diabetes is feasible, acceptable but associated with increased short-term anxiety: a randomized controlled trial in British general practice BMC Public Health 2008 14 350 doi:10.1186/1471-2458/8/350 10.1186/1471-2458-8-350 18840266 PMC2567326

[B32] McKenzie SH Jayasinghe UW Fanaian M Passey M Lyle D Powell Davies G Harris MF Socio-demographic factors, behaviour and personality: associations with psychological distress Eur J Prev Cardiol 2011 14 250 257 doi:10.1177/1741826711399426 21450585 10.1177/1741826711399426

[B33] Ansseau M Fischler B Dierick M Albert A Leyman S Mignon A Socioeconomic correlates of generalised anxiety disorder and major depression in primary care Depress Anxiety 2008 14 506 513 10.1002/da.20306 17595015

[B34] McHugh RK Daughters SB Lejuez CW Murray HW Hearon BA Gorka SM Otto MW Shared variance among self-report and behavioral measures of distress intolerance Cogn Ther Res 2011 14 266 275 doi:10.1007/s10608-010-9295-1 10.1007/s10608-010-9295-1 PMC3721199 23894216

[B35] Begg S Vos T Barker B Stevenson C Stanley L Lopez AD The burden of disease and injury in Australia 2003. PHE 82 2007 Canberra: AIHW

[B36] Australian Institute of Health and Welfare Prevention of cardiovascular disease, diabetes and chronic kidney disease: targeting risk factors. PHE 118 2009 Canberra: AIHW

